# Supplementary material for: Impact of Different Cell Counting Methods in Molecular Monitoring of Chronic Myeloid Leukemia Patients
Source: Diagnostics (Basel). 2022 Apr 22;12(5):1051. doi: 10.3390/diagnostics12051051 (PMC9140187; doi:10.3390/diagnostics12051051)
Supplement: Supplementary file 1 [file diagnostics-12-01051-s001.zip › Supplemental Figures.pdf]

Supplemental Figures

Supplementary Figure S1. *Bland–Altman showing the concordance of the BCR-ABL1 CT values measured in matched samples counted by the manual and three automatic cell-counting assays.* Paired measurements of *BCR-ABL1* CT value were combined for patients stratified in three groups, each consisting of 30 individuals: Group A (10% >BCR-ABL/ABLIS >1%) (**A - C**), Group B (1% >BCR-ABL/ABLIS>0.1%) (**D - F**), and Group C (0.1% >BCR-ABL/ABLIS >0.01%) (**G - H**). In each group of patients, differences in matched measurement are showed as comparison between the manual method with: the automatic assay using counting beads, the automatic protocol with 7-Aminoactinomycin D (7AAD) solution, and the automatic method based on the use of either counting beads or 7AAD. The graph is plotted on the XY axis where X depict the difference of the two measurements, and the Y-axis shows the mean of the two measurements. Horizontal lines are drawn at the mean difference between the two counting methods and the upper and lower limits of agreement. The 95% confidence intervals are shown for the mean and the upper and lower limits of agreement.

Supplementary Figure S2. *Bland–Altman showing the concordance of the ABL1 CT values measured in matched samples counted by the manual and three automatic cell-counting assays.* Paired measurements of *ABL1* CT value were combined for patients stratified in three groups, each consisting of 30 individuals: Group A (10% >BCR-ABL/ABLIS >1%) (**A - C**), Group B (1% >BCR-ABL/ABLIS>0.1%) (**D - F**), and Group C (0.1% >BCR-ABL/ABLIS >0.01%) (**G - I**). In each group of patients, differences in matched measurement are showed as comparison between the manual method with: the automatic assay using counting beads, the automatic protocol with 7-Aminoactinomycin D (7AAD) solution, and the automatic method based on the use of either counting beads or 7AAD. The graph is plotted on the XY axis where X depict the difference of the two measurements, and the Y-axis shows the mean of the two measurements. Horizontal lines are drawn at the mean difference between the two counting methods and the upper and lower limits of agreement. The 95% confidence intervals are shown for the mean and the upper and lower limits of agreement.
