# Supplementary figures and images for: Impact of Different Cell Counting Methods in Molecular Monitoring of Chronic Myeloid Leukemia Patients
Source: Diagnostics (Basel). 2022 Apr 22;12(5):1051. doi: 10.3390/diagnostics12051051 (PMC9140187; doi:10.3390/diagnostics12051051)

A

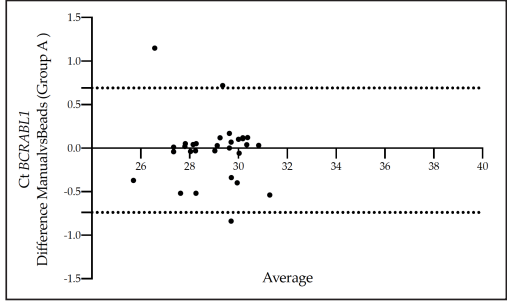

B

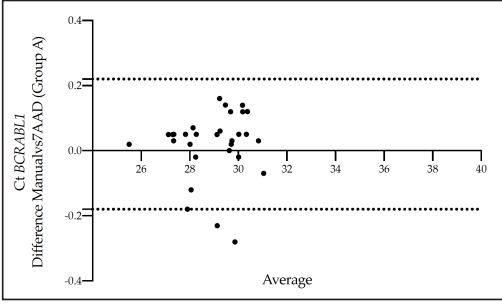

C

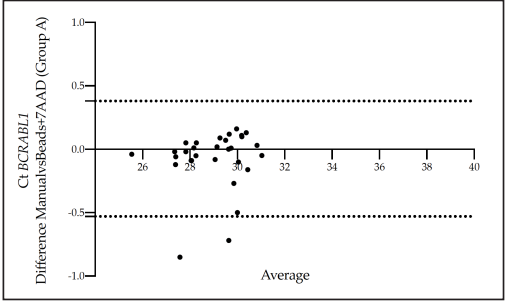

D

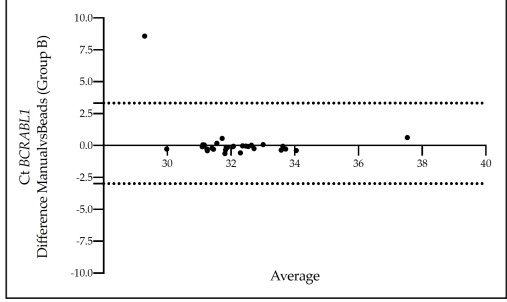

E

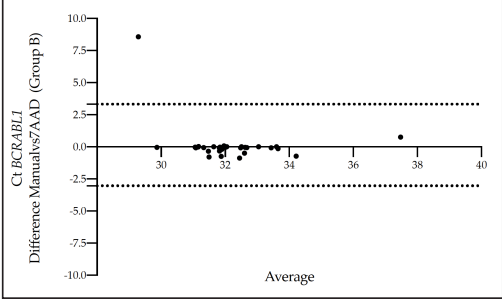

F

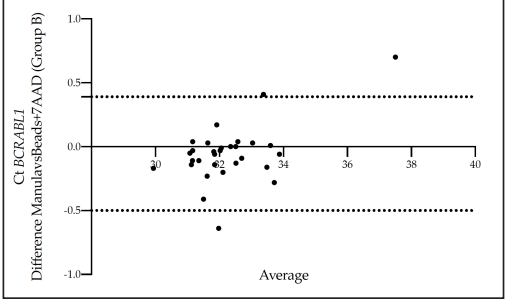

G

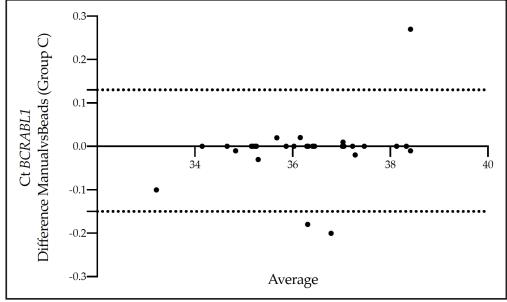

H

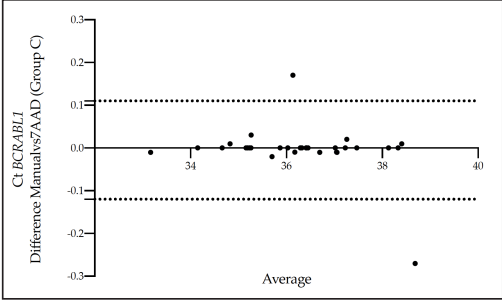

I

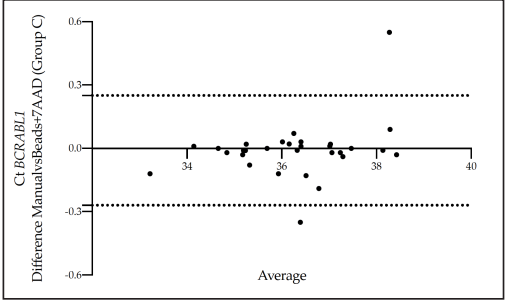

Supplement: Supplementary file 1 [file diagnostics-12-01051-s001.zip › Supplemental figure S1.pdf]

A

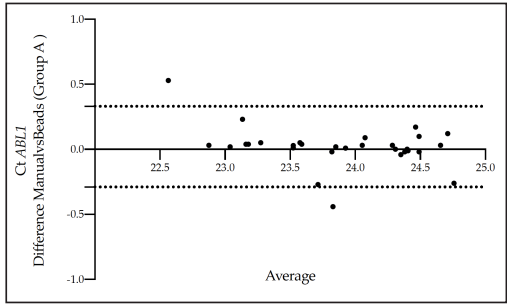

B

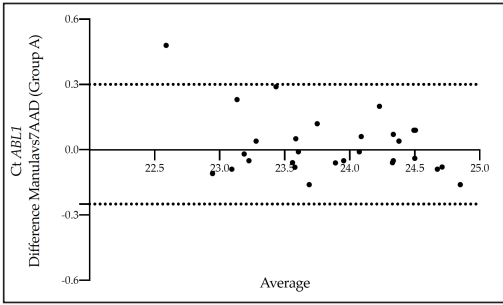

C

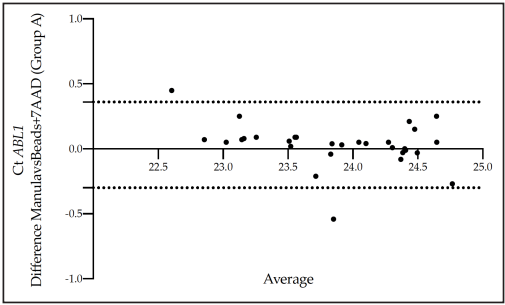

D

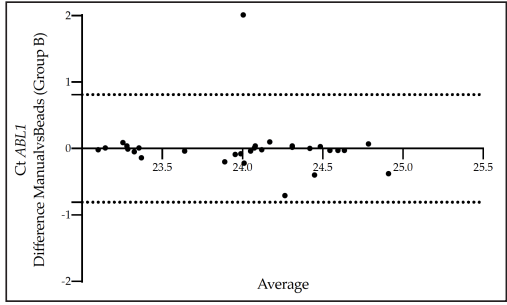

E

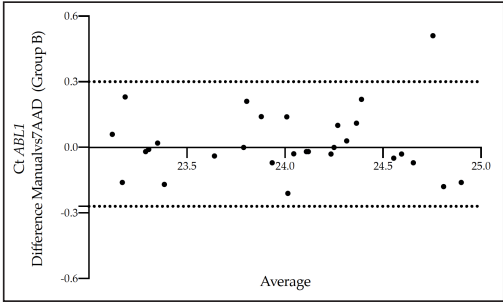

F

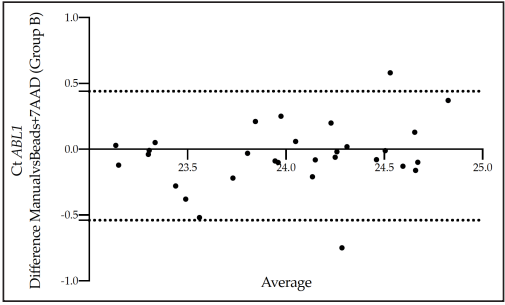

G

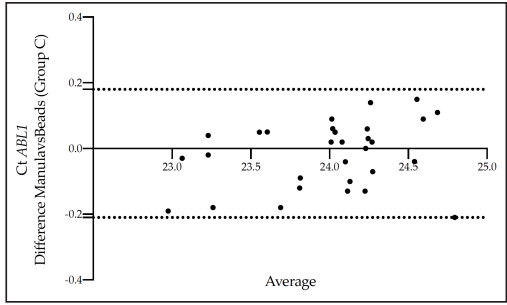

H

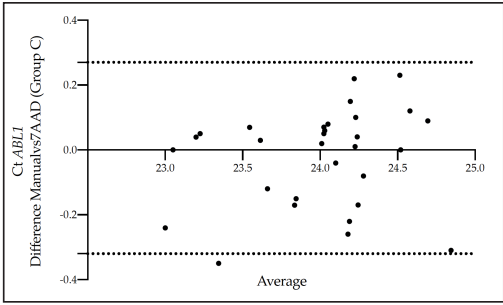

I

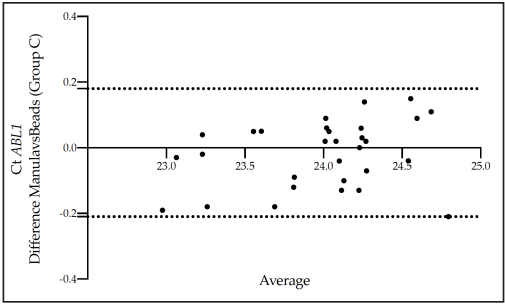

Supplemental Figure S2

Supplement: Supplementary file 1 [file diagnostics-12-01051-s001.zip › Supplemental figure S2.pdf]
